# Personalized Piperacillin Dosing for the Critically Ill: A Retrospective Analysis of Clinical Experience with Dosing Software and Therapeutic Drug Monitoring to Optimize Antimicrobial Dosing

**DOI:** 10.3390/antibiotics10060667

**Published:** 2021-06-03

**Authors:** Ute Chiriac, Daniel C. Richter, Otto R. Frey, Anka C. Röhr, Sophia Helbig, Judit Preisenberger, Stefan Hagel, Jason A. Roberts, Markus A. Weigand, Alexander Brinkmann

**Affiliations:** 1 Department of Pharmacy, Heidelberg University Hospital, 69120 Heidelberg, Germany; 2 Department of Anesthesiology, Heidelberg University Hospital, 69120 Heidelberg, Germany; Markus.Weigand@med.uni-heidelberg.de; 3 Department of Clinical Pharmacy, Heidenheim Hospital, 89522 Heidenheim, Germany; otto.frey@kliniken-heidenheim.de (O.R.F.); Anka.Roehr@Kliniken-Heidenheim.de (A.C.R.); sophia.helbig@kliniken-heidenheim.de (S.H.); judit.preisenberger@kliniken-heidenheim.de (J.P.); 4 Institute for Infectious Diseases and Infection Control, Jena University Hospital, 07740 Jena, Germany; stefan.hagel@med.uni-jena.de; 5 University of Queensland Centre for Clinical Research, Faculty of Medicine, The University of Queensland, Brisbane 4072, Australia; j.roberts2@uq.edu.au; 6 Departments of Pharmacy and Intensive Care Medicine, Royal Brisbane and Women’s Hospital, Brisbane 4072, Australia; 7 Division of Anaesthesiology Critical Care Emergency and Pain Medicine, Nîmes University Hospital, University of Montpellier, 30029 Nîmes, France; 8 Department of Anesthesiology, Heidenheim Hospital, 89522 Heidenheim, Germany; Alexander.Brinkmann@Kliniken-Heidenheim.de

**Keywords:** piperacillin, continuous infusion, therapeutic drug monitoring, dosing software, PK/PD

## Abstract

Optimization of antibiotic dosing is a treatment intervention that is likely to improve outcomes in severe infections. The aim of this retrospective study was to describe the therapeutic exposure of steady state piperacillin concentrations (c_PIP_) and clinical outcome in critically ill patients with sepsis or septic shock who received continuous infusion of piperacillin with dosing personalized through software-guided empiric dosing and therapeutic drug monitoring (TDM). Therapeutic drug exposure was defined as c_PIP_ of 32–64 mg/L (2–4× the ‘MIC breakpoint’ of *Pseudomonas aeruginosa*). Of the 1544 patients screened, we included 179 patients (335 serum concentrations), of whom 89% achieved the minimum therapeutic exposure of >32 mg/L and 12% achieved potentially harmful c_PIP_ > 96 mg/L within the first 48 h. Therapeutic exposure was achieved in 40% of the patients. Subsequent TDM-guided dose adjustments significantly enhanced therapeutic exposure to 65%, and significantly reduced c_PIP_ > 96 mg/L to 5%. Mortality in patients with c_PIP_ > 96 mg/L (13/21; 62%) (OR 5.257, 95% CI 1.867–14.802, *p* = 0.001) or 64–96 mg/L (30/76; 45%) (OR 2.696, 95% CI 1.301–5.586, *p* = 0.007) was significantly higher compared to patients with therapeutic exposure (17/72; 24%). Given the observed variability in critically ill patients, combining the application of dosing software and consecutive TDM increases therapeutic drug exposure of piperacillin in patients with sepsis and septic shock.

## 1. Introduction

Empirical antimicrobial therapy needs to be administered in a timely manner to critically ill patients with sepsis and septic shock to reduce mortality [1,2]. Furthermore, achievement of effective serum concentrations is important within the first 24–48 h of treatment [3]. However, optimal drug exposure is a difficult to achieve target and requires an understanding of both the pharmacokinetic (PK) changes that can occur in septic patients [4,5,6,7], and the antimicrobial pharmacodynamics (PD) [8] of the antimicrobial agent prescribed. Using the standard dosing regimen according to the Product Information is considered to have limited value in critical care as patients with sepsis and septic shock are likely to exhibit profound PK changes [9]. There is a strong body of evidence demonstrating that standard dosing approaches are concomitant with suboptimal plasma concentrations [9,10] leading to either inadequate antimicrobial exposure at the site of infection [11,12] or toxic serum concentrations [13,14,15,16] in a large fraction of critically ill patients. Therefore, antibiotic dosing in critically ill patients is very challenging, but should be considered a key intervention to further improve infection-related outcomes in patients with sepsis [17]. To this end, a more personalized approach to drug dosing, which understands PK in the individual patient and pathogen susceptibility (minimal inhibitory concentration (MIC); epidemiological cutoff (ECOFF)), should be further investigated [18].

In the case of ß-lactams, prolonged infusion is advantageous as it produces and maintains higher ß-lactam concentrations above the MIC, while intermittent infusion leads to unnecessary high peaks followed by low concentrations for a considerable part of the dosing interval [19,20,21]. Recent clinical data demonstrated that critically ill patients benefit from considerably higher (e.g., 1–4× MIC) and longer (e.g., 100% *f*T_>MIC_) ß-lactam exposures [22,23] than those described in earlier preclinical infection models [8]. Therapeutic drug monitoring (TDM) provides a robust method to ensure optimal exposures [24,25]. The use of dosing nomograms or dosing software [5,26,27] with respect to pathophysiological changes that occur in severe infections, sepsis and septic shock, respectively (i.e., renal function, renal replacement therapies (RRT) [28]) enables individual empiric dosing already in the early phase of treatment. Therefore, combining prolonged/continuous infusion with personalized empiric dosing and TDM is expected to be the safest and most effective way to ensure therapeutic drug exposure in critically ill patients [3,29,30]. 

The aim of this study was to describe the therapeutic exposure of piperacillin concentrations (c_PIP_) and clinical outcome in critically ill patients with a personalized dosing strategy including dosing software and TDM.

## 2. Results

In total, 1544 patients were admitted to the intensive care unit (ICU), of which 179 patients received empirical piperacillin/tazobactam treatment and were included. Moreover, in total, 335 piperacillin concentrations were available with each patient contributing 1 to 6 observations. Detailed demographic characteristics are summarized in Table 1 and Table 2. Diagnosis and bacterial pathogen distributions are presented in Table S1 in the supplement. Briefly, the study population was relatively old (median age 75 years) with moderate renal function (median 47 CrCL mL/minute) on admission. Overall, 33 (19%) patients received RRT. Of these patients, 18 (11%) were treated with continuous renal replacement therapy (CRRT) and 15 (8%) were treated with intermittent hemodialysis (iHD). The median (IQR) piperacillin daily dose was 8000 (7000) mg (software-guided empiric daily dose: 8000 (6750) mg, TDM-guided daily dose: 7000 (6675) mg), and thus less than the predicted median (IQR) piperacillin standard daily dose in these patients (12,000 (8000) mg). The median (IQR) c_PIP_ was 54 (34) mg/L (c_PIP_ after software-guided empiric daily dosing: 64 (38) mg/L, c_PIP_ after TDM-guided daily dosing: 49 (26) mg/L). 

### 2.1. Therapeutic Exposure

With software-guided empiric dosing, the minimum therapeutic exposure of >32 mg/L was overall realized in 89% of patients after empiric dosing including 22 patients (12%) with potentially harmful concentrations (c_PIP_ > 96 mg/L). Therapeutic exposure of 32–64 mg/L was observed in 72 patients (40%) within 48 h after onset of treatment. Subsequent TDM-guided dose adjustments significantly enhanced the target exposure of piperacillin concentrations to 65%, and significantly reduced c_PIP_ > 96 mg/L to 5%. With standard dosing, therapeutic exposure would have been realized in 76 patients (23%) whereas 125 patients (38%) would have shown c_PIP_ > 96 mg/L. The effect of personalized empiric dosing on therapeutic exposure is shown in Table 3. Data describing the distribution of c_PIP_ for all concentrations are shown in the supplement in Table S2 and for concentrations under CRRT in Table S3.

### 2.2. Predictors for Clinical Outcome

**Figure 1:**
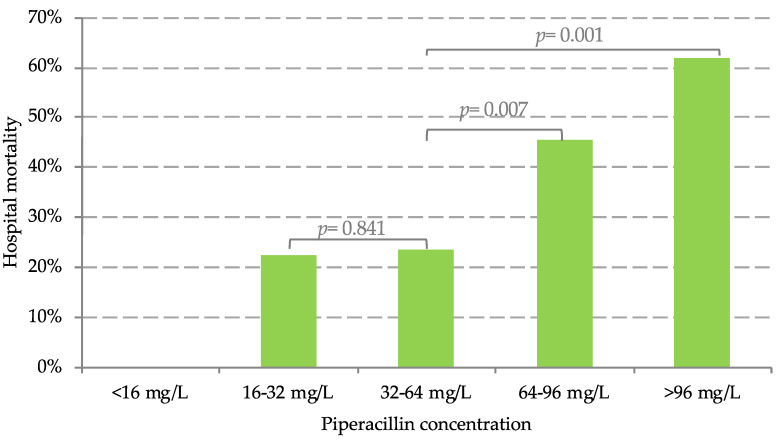
Distribution of hospital mortality rates. Hospital mortality rates over piperacillin concentration within 48 h after onset of treatment. Statistical analysis was performed using the chi-square test. Significant levels were considered as *p* ≤ 0.05.

The binary logistic regression revealed a high sequential organ failure assessment (SOFA) score (OR 3.533, 95% CI 1.993–6.262, *p* = 0.001), low creatinine clearance (CrCL) (OR 0.533, 95% CI 0.296–0.961, *p* = 0.036), and reduced piperacillin clearance (CL_PIP_) (OR 0.336, 95% CI 0.116–0.974, *p* = 0.045) to significantly increase the odds for hospital mortality within this study. There was no significant association with c_PIP_, age or BMI. Furthermore, mortality in patients with a c_PIP_ > 96 mg/L (13/21; 62%) (OR 5.257, 95% CI 1.867–14.802, *p* = 0.001) or in patients with c_PIP_ of 64–96 mg/L (30/76; 45%) (OR 2.696, 95% CI 1.301–5.586, *p* = 0.007) was significantly higher compared to patients with a piperacillin concentration of 32–64 mg/L (17/72; 24%). Mortality in patients with c_PIP_ ≤ 32 mg/L (4/19; 22%) (OR 1.159, 95% CI 0.339–3.965, *p* = 0.814) and patients with c_PIP_ of 32–64 mg/L (17/72; 24%) did not significantly differ (Table 4, Figure 1). Differences in median SOFA score and median simplified acute physiology score (SAPS) were not significant between patients with therapeutic drug exposure and patients with c_PIP_ > 64 mg/L (Table 4). 

**Figure 2:**
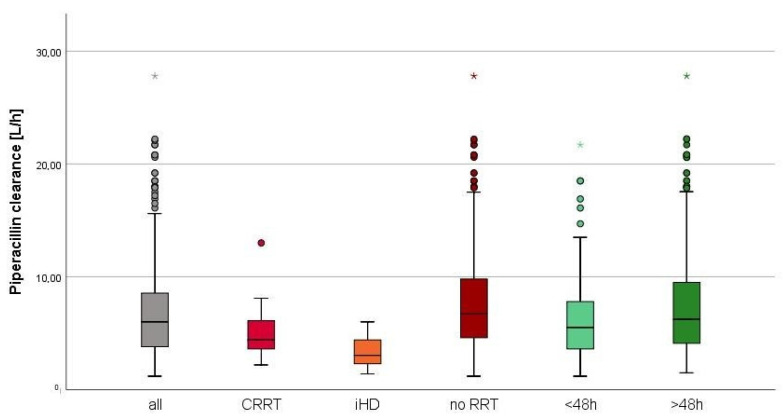
Observed piperacillin clearance. Distribution of the observed piperacillin clearance given in L/h of all patients (*n* = 335), patients with CRRT (*n* = 35), patients with iHD (*n* = 42), patients without RRT (*n* = 258), in the first 48 h (*n* = 179) and after 48 h (*n* = 156).

**Figure 3:**
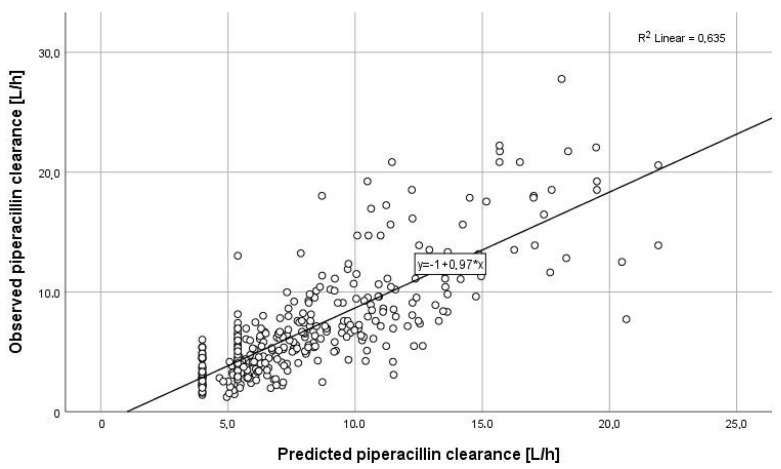
Piperacillin clearance predicted by CADDy versus observed piperacillin clearance within the first 48 h (r^2^ = 0.635). The solid black line shows the linear regression line of fit. The estimates of bias and precisions were also acceptable (0.30 and 1.30).

The median observed piperacillin clearance (CL_PIP_) was 6.7 (5.4) L/h (Figure 2). In the first 48 h, no augmented clearance was observed (Figure 2). The median piperacillin clearance predicted by CADDy (CL_CADDy_) was higher compared to the observed clearance (CL_PIP48_) in the first 48 h (CL_CADDy_ 8.3 (5.0) L/h vs. CL_PIP48_ 6.4 (5.3) L/h). The predictive capacity of CADDy was assessed by comparing the predicted with the observed clearance (Figure 3). The estimates of bias and imprecisions were also acceptable (0.30 and 1.30). 

## 3. Discussion

The present retrospective study evaluated the therapeutic exposure of piperacillin concentrations using a personalized dosing strategy including dosing software and consecutive TDM as recommended in the current guidelines [3,29,31,32]. Therapeutic exposure of piperacillin was high with a personalized dosing strategy. Already with empiric dosing guided by the Calculator to Approximate Drug-Dosing in Dialysis (CADDy) software the minimum therapeutic exposure of >32 mg/L was realized in 89% of the study population in the first 48 h. We identified 12% of the patients with potentially harmful c_PIP_ > 96 mg/L, which were reduced by 63% through subsequent TDM-guided dose adjustment. Furthermore, mortality in patients c_PIP_ >64 mg/L was significantly higher compared to patients with therapeutic exposure. Our data do not support previous findings of insufficiently low serum concentrations associated with continuous infusion of ß-lactams [9,13]. 

Sepsis and septic shock continue to be associated with high mortality [33,34]. The SOFA score and SAPS are established to predict the clinical outcomes in these patients [35,36]. The observed hospital mortality of sepsis (36%) and severe sepsis/septic shock (43%) is comparable to other findings in European ICUs reporting a mortality rate between 36.3 and 42.8% [37,38,39]. Critically ill patients with severe sepsis/septic shock in a large, prospective multicenter study in German ICUs also showed a hospital mortality rate of 40% [34]. 

From a PK point of view, a positive clinical outcome in critically ill patients is associated with increasing 100% *f*T_>MIC_ ratios [3]. Traditional antibiotic dosing repeatedly failed to attain this PK target recommendation in previous studies with critically ill patients. In the DALI trial, a prospective multinational study that included 361 critically ill patients who were treated with a β-lactam, only 30 and 67% of the patients achieved 100% *f*T_>4× MIC_ and 100% *f*T_>MIC_, respectively [9]. These findings were recently confirmed in the EXPAT study by Abdulla et al. [10]. Abdulla et al. found, that 37 and 63% of the patients with intermittent bolus application of β-lactams achieved 100% *f*T_>4× MIC_ and 100% *f*T_>MIC_, respectively [10]. It is of utmost importance to interpret the DALI and EXPAT data in the setting of the antibiotic application mode, since most patients in the DALI study and all patients in the EXPAT trial received an intermittent bolus application. With a personalized dosing approach including continuous infusion in this study, 49 and 99% of the patients achieved 100% *f*T_>4× MIC_ and 100% *f*T_>MIC_, respectively, within the first 48 h after onset of treatment. Only 10% of the patients demonstrated piperacillin concentrations below therapeutic exposure, 1% of the patients demonstrated piperacillin concentrations below the ECOFF of *Pseudomonas aeruginosa*. 

Using software-guided empiric dosing in the patients of this study demonstrated that an unnecessary high c_PIP_ (>96 mg/L) in only 12% of patients within the first 48 h after onset of treatment compared to 44% of patients would have exhibited c_PIP_ >96 mg/L in the case of standard doses administered as continuous infusion. Compared to these findings, a previous retrospective study from Richter et al. [14] found a very high number of patients receiving continuous infusion of piperacillin with potentially harmful concentrations. About 30% of the patients demonstrated c_PIP_ >100 mg/L within the first 48 h [14]. A possible explanation might be the implementation of empiric dose adjustment according to renal function rather than standard doses before the concentration measurement to avoid a high concentration either to high doses or decreased drug clearance. Harmful effects potentially related to excessively high serum concentrations (>96 mg/L) have been reported. Quinton et al. [15] observed neurotoxicity in about 40% of critically ill patients treated with a median daily dose of 12,000 mg (range 8000–12,000 mg) piperacillin administered by continuous infusion. The median serum concentration after 48 h was significantly higher compared to patients without neurotoxic symptoms (157 [95–236] mg/L vs. 91 [69–127] mg/L). Moreover, patients with neurotoxic C_PIP_ showed a remarkable reduction in CrCL within 48 h (46%). 

Due to the fact that ß-lactam antibiotics have the potential to precipitate antibiotic-induced toxicity is increasingly apparent [16], but likely remains underestimated in the clinical practice of sepsis therapy [16]. Dose-induced toxicity may manifest in the form of neurological deterioration and acute kidney injury (AKI). Acute kidney injury is a known major complication of vancomycin treatment, especially when it is co-administered with other nephrotoxins. A combination therapy of vancomycin with piperacillin/tazobactam is associated with higher acute kidney injury rates than its parallel use with meropenem or cefepime [40,41]. In a recent clinical study, Dhaese et al. identified a significantly higher mortality in critically ill patients with piperacillin concentrations of 64–160 mg/L [13]. Scharf et al. found no benefit for patients who reached the highest target of 100% *f*T_>4x MIC_, but a significant higher mortality rate [23]. Although confounding factors such as worse liver function, higher APACHE II, and SOFA scores must be considered, the authors recommended a PK target of 100% *f*T_>1x MIC<4x MIC_ for critically ill patients. In our study, hospital mortality was significantly higher in patients with concentrations above therapeutic exposure. In patients with c_PIP_ > 96 mg/L (corresponding 4–6x ECOFF *Pseudomonas aeruginosa*) hospital mortality was 5 times higher compared to patients with therapeutic exposure, despite the fact that the SOFA and SAPS scores were not significantly different in these patients. However, from these data it cannot be concluded that high levels are harmful or whether they are a prognostic factor. On the other hand, the benefit of high concentrations is debatable. There is only one in vitro infection model with piperacillin/tazobactam that reported a relationship between bacterial kill and %*f*T_>MIC_ with significant thresholds of 27% for bacteriostasis and 75% for bactericidal activity [42]. 

Considering the association of potentially toxic serum concentration (*f*T_>4× MIC_) and a higher mortality [13,14,23], this study strongly supports the present recommendations on personalized antibiotic dosing [3,18,31,43] including continuous application of ß-lactams [20,21,31,32] in critically ill patients with sepsis and septic shock. Hydrophilic β-lactam antibiotics demonstrate a foremost renal elimination. Consequently, renal function is the most relevant covariate for individualization of β-lactam dosing to achieve therapeutic exposure [44]. In patients with impaired renal function, the relationship between decreased piperacillin clearance and increased concentration, more specifically decreased drug amount, may not come as a surprise as previous studies have already demonstrated the correlation between CrCL and clearance of β-lactam antibiotics [9,18,24,45]. In line with previous studies, we identified pathophysiological changes presented as reduced piperacillin clearance compared to non-critically ill patients [14]. Piperacillin clearance demonstrated a high variability. However, previous findings frequently postulated a normal ß-lactam clearance independent of the renal function in critically ill patients in the beginning of the infection resulting in underdosing within the first 48 h after onset of treatment [13,15]. Our data do not support these considerations and suggest a dose adjustment according to renal function from the beginning to avoid very high piperacillin concentrations. With a median age of 75 years and a compromised renal function (median CrCL 47 mL/min) by the time of admission the investigated study cohort resembles a realistic cross section of patients treated in interdisciplinary ICUs [34]. 

Therefore, an optimal dosing scheme that is worthy of implementation into clinical practice as an optimal renal dose adjustment should balance the probability of achieving therapeutic exposure against the risks of toxicity and the emergence of antimicrobial resistance [46,47]. Given the observed variability in critically ill patients, the dose adjustment according to renal function using, for example, the dosing software might help avoid potentially harmful effects of very high ß-lactam concentrations, in particular in patients with compromised renal function [18,43]. Further data on the benefit of TDM-based dose optimization in septic patients with piperacillin treatment are expected soon in the already completed TARGET trial [48].

The present study has several limitations. First, the study was a single center study which may have hampered robust estimates of the extent of PK variability. Second, CrCL was estimated using the Cockcroft-Gault equation since the CrCL measurement is not performed in routine clinical care and the ß-lactam clearance and Cockcroft-Gault equation show a good overall correlation (r = 0.57) [14,49]. 

Third, non-renal clearance and organ dysfunctions (other than renal) might be relevant but are not considered in the CADDy calculation. Active metabolites that might influence drug clearance, efficacy, and toxicity cannot be estimated by the algorithm. Although, the observed piperacillin clearance shows a good correlation to the CADDy-predicted piperacillin clearance. Finally, this was a retrospective analysis of serum concentrations measured as total drug concentrations. Therefore, drawing causal relations from retrospective data is hardly possible and mortality data should be interpreted carefully as biasing factors might be present. However, a retrospective analysis of serum concentrations has the advantage of considering all patients, even those with low survival rates and might show a more realistic picture compared to prospective approaches. 

## 4. Material and Methods

### 4.1. Study Design and Population

This was a retrospective observational study at a German academic teaching hospital in 2013. Ethical approval was waived by the Ethics Commission of the University of Ulm, Germany (project number 137/19). All critically ill patients admitted to the ICU were screened and patients > 18 years of age, with sepsis, severe sepsis or septic shock (according to the meanwhile revised Surviving Sepsis Campaign (SSC) definition [50]) and empiric piperacillin/tazobactam treatment administered by continuous infusion were included in the study. Patients were excluded if they were <18 years of age or treated with piperacillin administered by intermittent infusion.

### 4.2. Study Procedures

**Figure 4:**
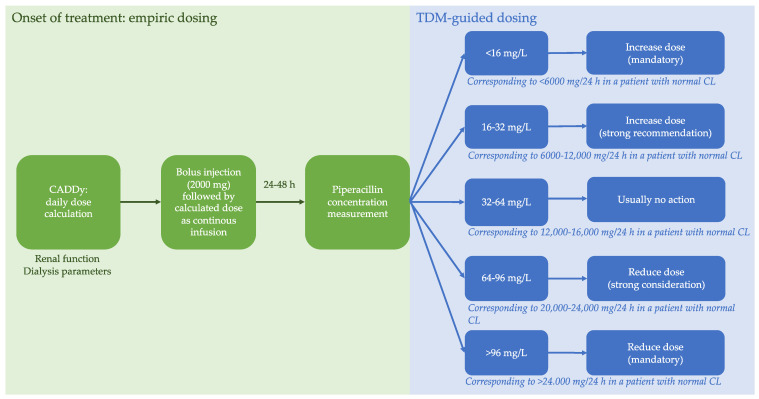
Dosing procedures. Therapeutic drug monitoring (TDM)-based dose adaptation strategy for continuous infusion. CADDy Calculator to Approximate Drug-Dosing in Dialysis; CL piperacillin clearance; TDM Therapeutic drug monitoring.

All patients received initial antibiotic treatment within the first 3 h after diagnosis according to the effective Surviving Sepsis Campaign (SSC) guideline of 2013 [50]. Piperacillin was administered by TDM-guided continuous infusion in all patients according to our standard operating procedure [51]. This approach consisted of a loading dose (2000 mg, 15 min infusion) followed by immediate continuous infusion with an empiric dose using the calculator to approximate drug-dosing in dialysis (CADDy) and subsequently adjusted by TDM within the first 24–48 h (Figure 4). The CADDy program (www.thecaddy.de accessed on 1 January 2013; Dr. Otto Frey, Klinikum Heidenheim) was used to predict piperacillin clearance (CL_CADDy_) as well as piperacillin doses in patients considering the CrCL evaluated by the Cockcroft-Gault equation, and if applicable dialysis settings [5]. TDM-guided dose adjustments and consecutive TDMs were advised and supervised by trained clinical pharmacists. Piperacillin concentration was measured, using a validated high-performance liquid chromatography assay (HPLC) [52]. 

### 4.3. Assessment of Therapeutic Drug Exposure

A therapeutic drug exposure was defined as c_PIP_ of 32–64 mg/L corresponding to two to four times the ‘MIC breakpoint’ of *Pseudomonas aeruginosa* (http://www.eucast.org/clinical_breakpoints: Piperacillin 16 mg/L, accessed on 1 January 2013) as a rational compromise between effective bacterial killing and possible harmful concentrations. Piperacillin concentrations of 64–96 mg/L were defined as moderately high. To assess the suitability of empiric dosing considering individual pharmacokinetics, concentration was stratified by the time of observation and prediction was evaluated. In addition, empiric dosing was compared to a continuous infusion of standard doses according to the national committee on antimicrobial susceptibility testing in Germany (NAK). Concentrations were predicted by the observed CL_PIP_. A one-compartment model was used to perform PK analyses since piperacillin has a small volume of distribution, low protein binding, and is essentially excreted by the kidneys. CL_PIP_ was calculated using the following equation: ${\mathrm{CL}}_{\mathrm{PIP}}\left[L/h\right]=\frac{\mathrm{dose}\left[\mathrm{mg}\right]\;}{24h}\cdot c_{\mathrm{PIP}}{}^{-1}\;\left[\mathrm{mg}/L\right]$.

Distribution of clinical parameters was assessed in relation to c_PIP_ and factors likely to contribute to clinical outcome were analyzed for association based on clinical relevancy or previously described relationships [53,54,55]. These included patient characteristics (age, BMI), illness severity scores (SOFA), cPIP, serum creatinine, eGFR, and CL_PIP_.

### 4.4. Statistical Analysis

All calculations and statistical analysis were performed using IBM SPSS Statistics version 26 software (IBM, Armonk, NY, USA). Discrete variables are expressed as counts (percentage) and continuous variables as means ± standard deviation (SD) or median with the interquartile range (IQR). We used the logistic regression analysis to evaluate the association of clinical outcomes with patient characteristics. Differences between groups were assessed for statistical significance using the Kruskal–Wallis test for continuous variables and the chi-square test or Fisher’s exact test for categorical variables. Significant levels were considered as *p* ≤ 0.05.

For pharmacokinetic analysis, we evaluated the predicted clearance against the observed clearance using the ratio of predicted and observed piperacillin concentrations (relative error). Bias was assessed by calculating the median of the relative errors of all observations and subtracting 1. Precision was assessed from the magnitude of the relative error. We chose to report median errors, as they were not normally distributed, according to the alternative method of Sheiner and Beal [56].

## 5. Conclusions

Our data strongly support the use of dosing interventions including the application of dosing software, CI, and TDM to ensure sufficient therapeutic drug exposure in septic patients with an *a priori* high mortality rate. Personalized dosing strategies lead to therapeutic drug exposure within the first 48 h of treatment as well as throughout the treatment course, while avoiding critically low piperacillin concentrations and minimizing potentially harmful piperacillin concentrations at the same time. CADDy reliably predicts the piperacillin clearance (empiric dosing) in critically ill patients. However, further validation of the dosing software in a clinical trial is required.

### Key Messages

- Piperacillin clearance in critically ill patients with septic patients shows high variability;
- Recommended personalized dosing strategy of piperacillin, including dosing software and TDM, ensures adequate serum concentrations;
- CADDy is a useful and reliable dosing software for empiric dose calculations.

## Figures and Tables

**Table 1:** Patient characteristics.

	Median (IQR), *n* (%)
Age, years	75 (15)
Weight, kg	78 (18)
Height, cm	170 (13)
BMI, kg/m^2^	26 (7)
Sex male	115 (64%)
Creatinine, mg/dL	1.18 (1.13)
CrCL, mL/min	47 (50)
RRT	33 (19%)
CRRT	18 (10%)
iHD	15 (8%)
Mechanical ventilation	105 (59%)
SOFA	6 (6)
SAPS	37 (18)
APACHE II *	22 (14)
ICU mortality	56 (31%)
Hospital mortality	64 (36%)
Length of hospital stay, days	17 (18)
Antimicrobial treatment, days	6 (4)

BMI: Body mass index; CrCL: Creatinine clearance; CRRT: Continuous renal replacement therapy; iHD: Intermittent hemodialysis; ICU: Intensive care unit; RRT: Renal replacement therapy; SAPS: Simplified acute physiology score; SOFA: Sequential organ failure assessment. * On day of inclusion.

**Table 2:** Diagnosis.

Diagnosis	*n* (%)
Sepsis/Severe sepsis	126 (70%)
Septic shock	53 (30%)
Site of infection	
Pneumonia	96 (54%)
Abdominal infection, peritonitis	37 (23%)
Soft tissue/bone infection	10 (6%)
Urinary tract infection	11 (7%)
Endocarditis, blood stream infection	7 (4%)
Cholecystitis, cholangitis	6 (4%)
Diverse	10 (6%)

Values are given in absolute numbers (*n*) and relative incidence (%).

**Table 3:** Effect of personalized empiric dosing on therapeutic exposure. Distribution of piperacillin concentrations (c_PIP_) in 179 critically ill patients with a continuous infusion personalized by the dosing software (=c_PIP_ observed based on software-guided empiric dosing) compared to a continuous infusion of standard doses according to national guidelines (NAK; National committee on antimicrobial susceptibility testing in Germany) (=c_PIP_ predicted based on standard dosing) within 48 h after the onset of treatment.

c_PIP_ (mg/L)	<16	16–32	32–64	64–96	>96
Predicted based on standard dosing	0 (0%)	3 (1.7%)	35 (19.6%)	63 (35.2%)	78 (43.6%)
Software-guided empiric dosing	1 (0.6%)	18 (10.1%)	72 (40.2%)	66 (36.9%)	22 (12.3%)

Values are given in absolute number (*n*) and relative incidence (%). c_PIP_: Piperacillin concentration; TDM: Therapeutic drug monitoring.

**Table 4:** Cross table depicting the distribution of clinical parameters in different piperacillin concentration (c_PIP_) groups.

c_PIP_ (mg/L)	<16	16–32	32–64	64–96	>96
Hospital mortality (%)	0%	22%	24%	45% *	62% *
Median SOFA score (IQR)	2 (1) *	3 (7) *	6 (6)	7 (8)	6 (5)
Median SAPS (IQR)	26 (7) *	30 (23) *	37 (19)	38 (20)	37 (7)
Median CL_PIP_ (L/h) (IQR)	11.5 (8.8)	12.8 (10.6)	6.9 (5.0) *	5.0 (3.2) *	2.8 (1.7) *
Median CrCL (mL/min) (IQR)	71.6 (12.6)	88.5 (91.4)	42.1 (54.1) *	38.7 (38.8) *	23.2 (19.1) *
Median age (years) (IQR)	77 (0)	61 (24)	72 (16)	77(13)	79 (11)
Median BMI (kg/m^2^) (IQR)	30 (0)	29 (8)	27 (7)	25 (7)	25 (5)

* *p* < 0.05. Values are given as median (IQR) or relative incidence (%). CrCL: Creatinine clearance; CL_PIP_: Observed piperacillin clearance; SAPS: Simplified acute physiology score; SOFA: Sequential organ failure assessment.

## Data Availability

The datasets used and analyzed during the current study are available from the corresponding author on reasonable request.

## Supplementary Materials

The following are available online at https://www.mdpi.com/article/10.3390/antibiotics10060667/s1, Table S1: Distribution of pathogens; Table S2: Piperacillin concentrations stratified by the time of observation; Table S3: Piperacillin concentrations under continuous renal replacement therapy.
