# Supplementary material for: Personalized Piperacillin Dosing for the Critically Ill: A Retrospective Analysis of Clinical Experience with Dosing Software and Therapeutic Drug Monitoring to Optimize Antimicrobial Dosing
Source: Antibiotics (Basel). 2021 Jun 3;10(6):667. doi: 10.3390/antibiotics10060667 (PMC8227218; doi:10.3390/antibiotics10060667)
Supplement: Supplementary file 1 [file antibiotics-10-00667-s001.zip › antibiotics-1223022-supplementary.pdf]

Table S1. Distribution of pathogens.

**Pathogens**	***n* (%)**
Escherichia coli, Proteus spp, *Klebsiella* spp	41 (23%)
ESBL Escherichia coli	4 (2%)
Enterobacter spp, *Citrobacter* spp, *Serratia* spp	12 (7%)
Pseudomonas aeruginosa, Acinetobacter spp	10 (6%)
MSSA	14 (8%)
CoNS	14 (8%)
Viridans group streptococci	8 (4%)
Enterococcus faecium	4 (2%)
Enterococcus faecalis	3 (2%)
Mixed gram +/−	2 (1%)
Other	2 (1%)
Candida spp	7 (4%)
No detection	58 (32%)

Values are given in absolute numbers (*n*) and relative incidence (%). CoNS: Coagulase-negative staphylococci; ESBL: Extended-spectrum ß-lactamase; MSSA: Methicillin-susceptible *Staphylococcus aureus.*

Table S2. Piperacillin concentrations stratified by the time of observation. Distribution of piperacillin concentrations (c_{PIP}) in critically ill patients with a personalized dosing strategy including the dosing software, continuous infusion, and therapeutic drug monitoring (TDM) within 48 h (=c_{PIP} based on software-guided empiric dosing) and after 48 h (=c_{PIP} based on TDM-guided dosing). Values are given in absolute and relative incidence.

**c_{PIP} (mg/L)**	**<16**	**16–32**	**32–64**	**64–96**	**>96**
Software-guided empiric dosing (*n* = 179)	1 (0.6%)	18 (10.1%)	72 (40.2%)	66 (36.9%)	22 (12.3%)
TDM-guided dosing (*n* = 156)	0 (0%)	27 (17.3%)	102 (65.4%)	20 (12.8%)	7 (4.5%)

Values are given in absolute number (*n*) and relative incidence (%). c_{PIP}: Piperacillin concentration; TDM: Therapeutic drug monitoring.

Table S3. Piperacillin concentrations under continuous renal replacement therapy. Distribution of piperacillin concentrations (c_{PIP}) in critically ill patients with continuous renal replacement therapy (CRRT) and a personalized dosing strategy including dosing software, continuous infusion, and therapeutic drug monitoring (TDM) within 48 h (=c_{PIP} based on software-guided empiric dosing) and after 48 h (=c_{PIP} based on TDM-guided dosing). Values are given in absolute and relative incidence.

**c_{PIP} (mg/L)**	**<16**	**16–32**	**32–64**	**64–96**	**>96**
Software-guided empiric dosing (*n* = 16)	0 (0%)	2 (12.5%)	5 (31.3%)	8 (50.0%)	1 (6.3%)
TDM-guided dosing (*n* = 17)	0 (0%)	4 (23.5%)	13 (68.4%)	2 (6.3%)	0 (0%)

Values are given in absolute number (*n*) and relative incidence (%). c_{PIP}: Piperacillin concentration; TDM: Therapeutic drug monitoring.
